# A preliminary comparative study of percutaneous CT-guided cryoablation with surgical resection for osteoid osteoma

**DOI:** 10.7717/peerj.10724

**Published:** 2021-01-15

**Authors:** Liangliang Meng, Xiao Zhang, Ruijiang Xu, Bin Wu, Xiaobo Zhang, Yingtian Wei, Jing Li, Husheng Shan, Yueyong Xiao

**Affiliations:** 1 Medical School of Chinese PLA, Beijing, China; 2 Department of Radiology, the First Medical Centre, Chinese PLA General Hospital, Beijing, China; 3 Department of Radiology, Chinese PAP Beijing Corps Hospital, Beijing, China; 4 Department of Pediatric Surgery, Chinese PLA General Hospital, Beijing, China

**Keywords:** Cryoablation, Osteoid osteoma, Surgery, Computed tomography

## Abstract

**Background:**

The traditional treatment for osteoid osteoma is the nidus’ surgical resection, which was difficult to eradicate with more invasive and complications because of osteosclerosis surrounding the nidus. This study aimed to analyze the efficacy and safety of percutaneous CT-guided cryoablation of osteoid osteoma at different sites (especially refractory sites such as the spine).

**Methods:**

Fifteen patients with osteoid osteoma who underwent cryoablation at our institution were analyzed retrospectively on their imaging data and clinical visual analog scale (VAS) pain scores before and after the procedure. Fifty-three patients underwent surgical resection during the period were also included in this study as a control group. Treatment efficacy was assessed primarily by comparing the differences in VAS scores at different time points in each group of patients by paired-sample t-test. Differences in length of hospital stay and complications between the two groups were also compared.

**Results:**

The technical success rate was 100% in both the cryoablation and surgical resection group. Cryoablation had a significantly shorter hospitalization time than surgery (*p* = 0.001). Clinically, the post-operative VAS scores were all significantly improved compared to the pre-operative period, and the clinical cure was achieved in both groups. Surgical operations had more complications than cryoablation, although there was no significant difference. In the group of cryoablation, only one patient had mild numbness of the left lower extremity, which relieved itself; two patients had mild post-operative pain. No patients in the cryoablation group experienced recurrence during the follow-up period. In the surgery group, three of the patients experienced massive bleeding (>500 ml), and two underwent transfusion therapy. Only one patient in the surgical resection group experienced a recurrence at 29 months postoperatively and underwent a second resection. All patients had local scars on the skin after surgical resection.

**Conclusion:**

Cryoablation is a minimally invasive, safe, and effective treatment strategy for osteoid osteoma, and is fully comparable to surgical resection.

## Introduction

Osteoid osteoma (OO) is a benign osteogenic tumor that develops mainly in the long tubular bones of the lower extremities and most commonly seen in children and adolescents (Atesok et al., 2011). The main clinical feature is marked nocturnal pain, which can be relieved by oral non-steroidal anti-inflammatory drugs (NSAIDs). The pathological feature shows a small and round nidus, which is composed of bone-like tissue and immature bone trabeculae, surrounded by sclerotic bone (Orth & Kohn, 2017). Removing of the nidus is the only curative treatment, although it is difficult to be entirely resected due to the surrounding sclerotic bone, and a recurrence would happen (Jannelli et al., 2020; Orth & Kohn, 2017; Payo-Ollero et al., 2020). Other shortages of surgical resection are its traumatic nature for adolescents, slower recovery, and the fact that complications such as fractures and joint dysfunction are reported (Payo-Ollero et al., 2020). In addition to surgical resection, CT-guided percutaneous drilling and resection (PDR) is another minimally invasive method that had been used frequently in clinical practice for the treatment of OO. Still, this method usually requires total anesthesia in patients and has a greater chance of post-operative recurrence (Agashe et al., 2020).

In recent years, with the development of imaging-guided minimally invasive techniques, radiofrequency, microwave, and laser ablation have been widely used in OO, with high accuracy, excellent therapeutic efficacy, and better safety (Ringe, Panzica & Falck, 2016; Takahashi & Berber, 2020; Tomasian & Jennings, 2019; Young, Golzarian & Anderson, 2019). Radiofrequency ablation is the most commonly used treatment for OO in minimally invasive interventions other than surgery (Lindquester, Crowley & Hawkins, 2020; Tordjman et al., 2020). Microwave ablation is another thermal ablation method that uses microwaves to generate heat through the vibration of polar water molecules in the target area. It has exhibited comparable results to radiofrequency ablation in OO treatment but has rarely been applied to the nidus located in the spine (De Filippo et al., 2018; Esteban Cuesta et al., 2018). Laser ablation has also shown promising results in treating OO in various locations (Tomasian et al., 2020). In the treatment of OO, cryoablation was less commonly used than radiofrequency or microwave ablation. Only a few studies have reported on the application of cryoablation for OO; however, all of these have shown encouraging results (Coupal et al., 2014; Santiago et al., 2018; Whitmore et al., 2016; Wu et al., 2011). Previous applications have focused on long tubular bones and other refractory areas such as the spine and around joints, which have been less reported (Santiago et al., 2018; Wu, Lu & Chen, 2017). Although radiofrequency and microwave ablation have been widely used, their heat conduction properties may cause pain during treatment. Cryoablation, however, can significantly reduce the pain of patients during the procedure. Cryoablation does not produce thermal radiation and is not likely to cause damage to the joint capsule, joint cartilage or dural sac. Previous studies have simply analyzed the efficacy of minimally invasive treatments, including radiofrequency ablation, without visually comparing them to conventional surgical resection. In this study, compared to surgery operation, we retrospectively analyzed the efficacy and safety of cryoablation to treat different sites of OO and share some of our techniques and experiences in targeting specific site tumors. With the current study, we found that cryoablation is a fully comparable treatment to surgical resection with less trauma and fewer complications.

## Materials & Methods

### Patients

The Chinese PLA general hospital granted ethical approval to carry out the study within its facilities. With our institutional ethics committee’s approval, fifteen patients with OO who underwent CT-guided percutaneous cryoablation in our hospital from June 2009 to July 2019 were retrospectively analyzed, and fifty-three patients who underwent a surgical operation during this period were also recruited in this study as a control group. The end of the follow-up period was 31 July 2020. Because of the study’s retrospective nature, informed consent was waived by the Ethics Committee of our institution. This paper does not contain any person’s data in any form. We reviewed the hospital records and radiographic data of these patients. Pre-operative preparation usually included a clinical history, physical examination, pre-operative imaging evaluation, and laboratory tests. All patients were evaluated before and after treatment using a visual analog scale (VAS) system. Inclusion criteria of cryoablation included age under 80 years, a single lesion less than 3 cm, absence of severe cardiopulmonary dysfunction, an absence of malignancy tumors and other underlying severe diseases, and no coagulation disorders, or no anticoagulant medications in the last one week. Inclusion criteria for patients who underwent surgical resection during the same period included a single lesion less than four cm, with the remaining criteria similar to those for cryoablation. All patients included in the analysis of this study were required to have a follow-up time of more than 12 months. Relevant clinical information and demographics for all patients were presented in Table 1.

**Table 1:** Patient clinical information and demographics for all patients.

	Percutaneous cryoablation (*n* = 15)	Surgical resection (*n* = 53)	*p*-value
Age (years) (range)	16.13 ± 11.32 (6–52)	15.15 ± 9.37 (3–50)	0.411
Gender			0.816
Male	10	37	
Female	5	16	
Nidus size (mm) (range)	14.65 ± 4.02 (8.6–23.1)	16.42 ± 7.78 (4–33)	0.398
Nidus location			
Vertebra	3	11	
Femur	7	29	
Acetabulum	1	1	
Tibia	2	8	
Iliac	1	0	
Patella	1	0	
Maxilla	0	1	
Heel bone	0	1	
Humerus	0	2	
Symptoms			
Night pain	13	42	
NSAID use	10	26	
Duration (months) (range)	12.53 ± 9.20 (2–36)	12.71 ± 11.34 (0.5-48)	
The basis for the diagnosis			
Pathologically confirmed	9	53	
Diagnosed by radiography	6	0	

### The procedures of cryoablation

A warm water circulation blanket is placed under the patient to keep him warm. Depending on the lesion’s location, the patient lies on the scanning bed and is given electrocardiographic monitoring, oxygenation, and blood pressure measurement. A pre-operative CT scan of the puncture site was routinely performed to determine the puncture point and puncture path. The vast majority of patients can undergo cryoablation under local anesthesia. Only a minority of younger patients who cannot tolerate pain require general anesthesia before ablation. After local disinfection of the skin of the puncture point, laying of the hole towel, 20 ml of 1% lidocaine for local anesthesia. An 11G coaxial T-Lok bone marrow biopsy needle system was used to penetrate the osteocortex and target the lesion with CT guidance. After the needle core was removed, a 17G cryoprobe was inserted through a coaxial sheath to the nidus of the lesion. After another CT scan to determine the location of the probe, cryotherapy was started with freezing for 10 min, thawing 3 min, and then repeated the treatment once more to achieve complete tumor inactivation. CT scans were performed at 5-minute intervals during the treatment to determine the ice ball’s location and extent. The CT scan was performed again after the probe was removed to confirm the treatment’s effectiveness and exclude complications, including bleeding.

For three patients with an OO of the spine, unlike previous approaches in the literature, we innovatively used another 19G fine needle, which was pushed into the bony spinal canal, and 50–100 ml filed gas were injected into the epidural space to separate the dural sac from the bony canal, thus protecting the dural sac and the spinal cord from freezing. This may facilitate a complete ablation of the nidus with minimizing damage to normal structures.

### The procedures of surgical resection

Surgical procedures for OO vary in different sites. Anesthesia options for patients undergoing surgical resection include general anesthesia and epidural anesthesia. The surgery’s primary purpose is to remove the tumor and the surrounding affected tissues and, if necessary, to fill the residual cavity with autogenous or allogeneic bone implants. Some patients require intraoperative placement of a metal internal fixation to prevent fracture. Drains were placed around the surgical area to drain fluid exudation or post-operative bleeding as appropriate.

### Effective evaluation

All patients completed VAS scores before and after cryoablation or surgery to assess the efficacy of the procedures. We obtained the above information through in-patient records and follow-up records with the patient’s consent. Post-treatment follow-up imaging by CT or MR was performed to evaluate the ablation outcome and exclude potential complications, including osteonecrosis and fractures. Long-term post-operative outcomes were obtained by telephone or outpatient follow-up for recurrence and pain evaluation with another VAS score (>12 months). Technical success was defined as completion of the cryoablation or surgical procedure according to a protocol established preoperatively. Clinical success was defined as resolution or near-resolution of symptoms based on the VAS rating system, both immediately (<7 days) after treatment and after long-term follow-up (>12 months).

### Statistical analysis

Statistical analysis was performed using SPSS software (IBM SPSS statistics version 25, Chicago, IL, USA). A Paired two-sample *T*-test was used to compare the differences in VAS scores before and after cryoablation or surgical resection. The statistical threshold was set at a *p*-value of less than 0.05. Two independent samples t-tests were used to compare differences in clinical information and outcomes related to patients in the cryoablation and surgical groups.

## Results

### Patient clinical information and demographics

**Figure 1:**
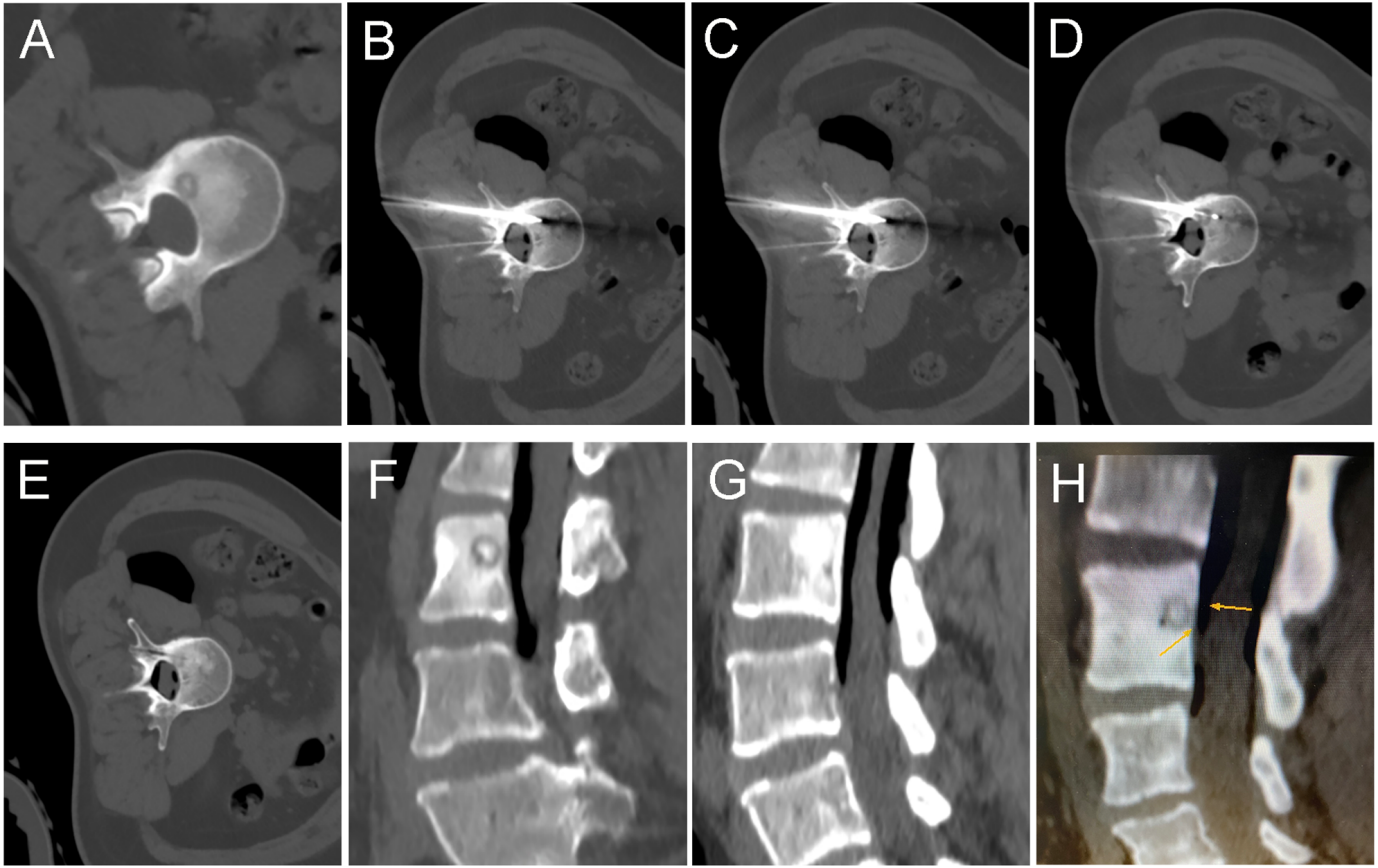
A 35-year-old female with intermittent low back pain for more than six months. (A) Pre-operative CT showed a round lower density lesion on the lumbar three vertebrae, with a dotted high-density nest in the center. (B, C, D) A cryoprobe was passed from the back through the appendages to reach the vertebral foci. At the same time, a 19G fine needle was used to puncture the epidural space, and a syringe was used to inject filed air into the spinal canal epidural space. (E) After the freezing was completed and the frozen needle was removed, a repeated CT scan should be performed to exclude complications. The needle path was visible. (F, G, H) Sagittal reconstruction images showed the vertebral body successfully isolated by air between the lesion and the dural sac. The air outside the dural sac protected the structures in the spinal canal from damage while freezing at full power.

**Figure 2:**
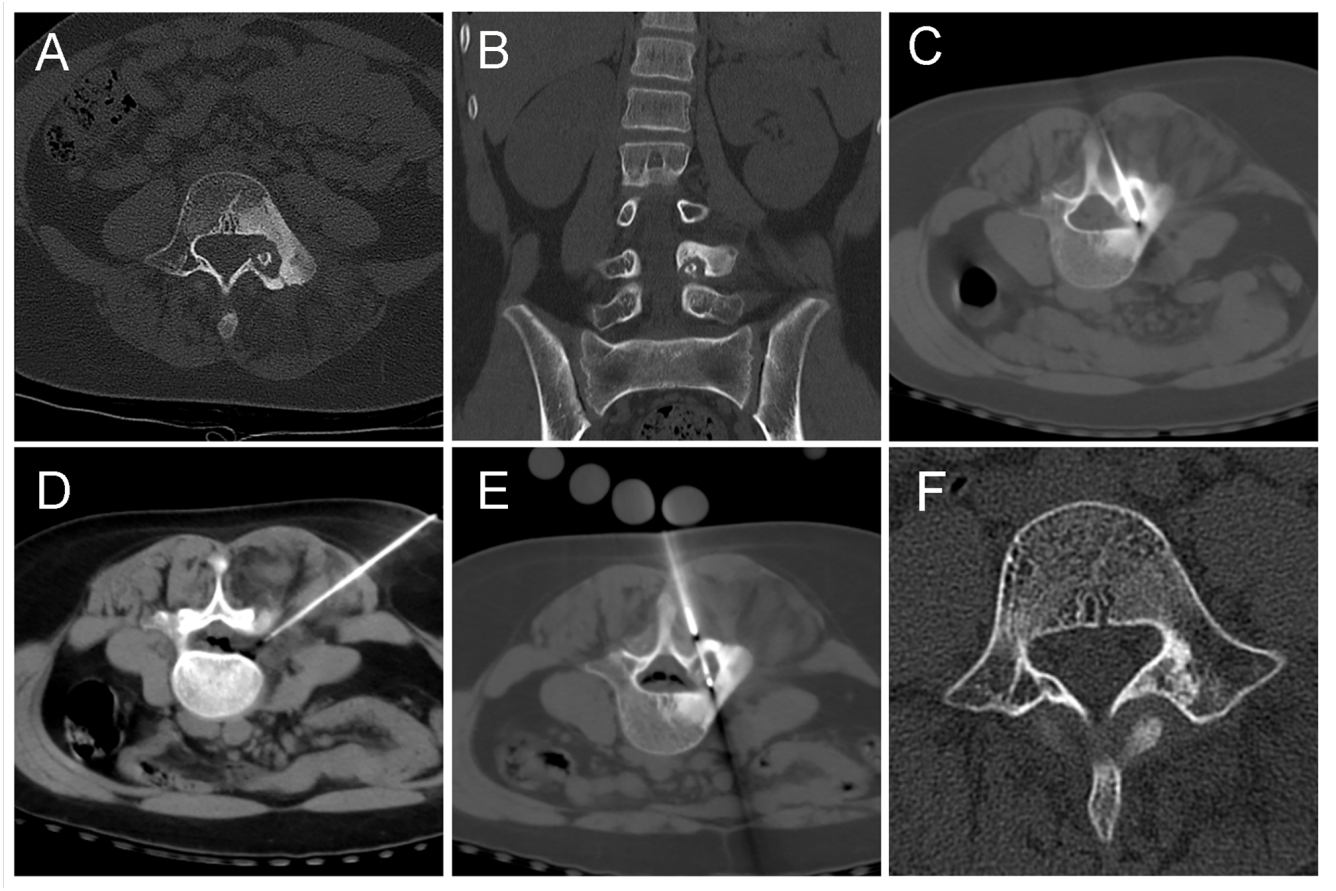
An 11-year-old girl with low back pain, predominantly at night. (A, B) The lesion was mainly located in the left pedicle with marked hyperplastic sclerosis of the bone around the lesion. (C, D, E) The patient was positioned prone, and the freezing probe was inserted from the back, directly through the center of the lesion. The fine needle was used to inject air into the epidural space to protect the intravertebral structures. (F) One year after the procedure, a follow-up CT scan showed that the former low-density lesion area had been replaced by high-density bone tissue, and the osteosclerotic area around the lesion had surprisingly returned to normal on this review.

**Figure 3:**
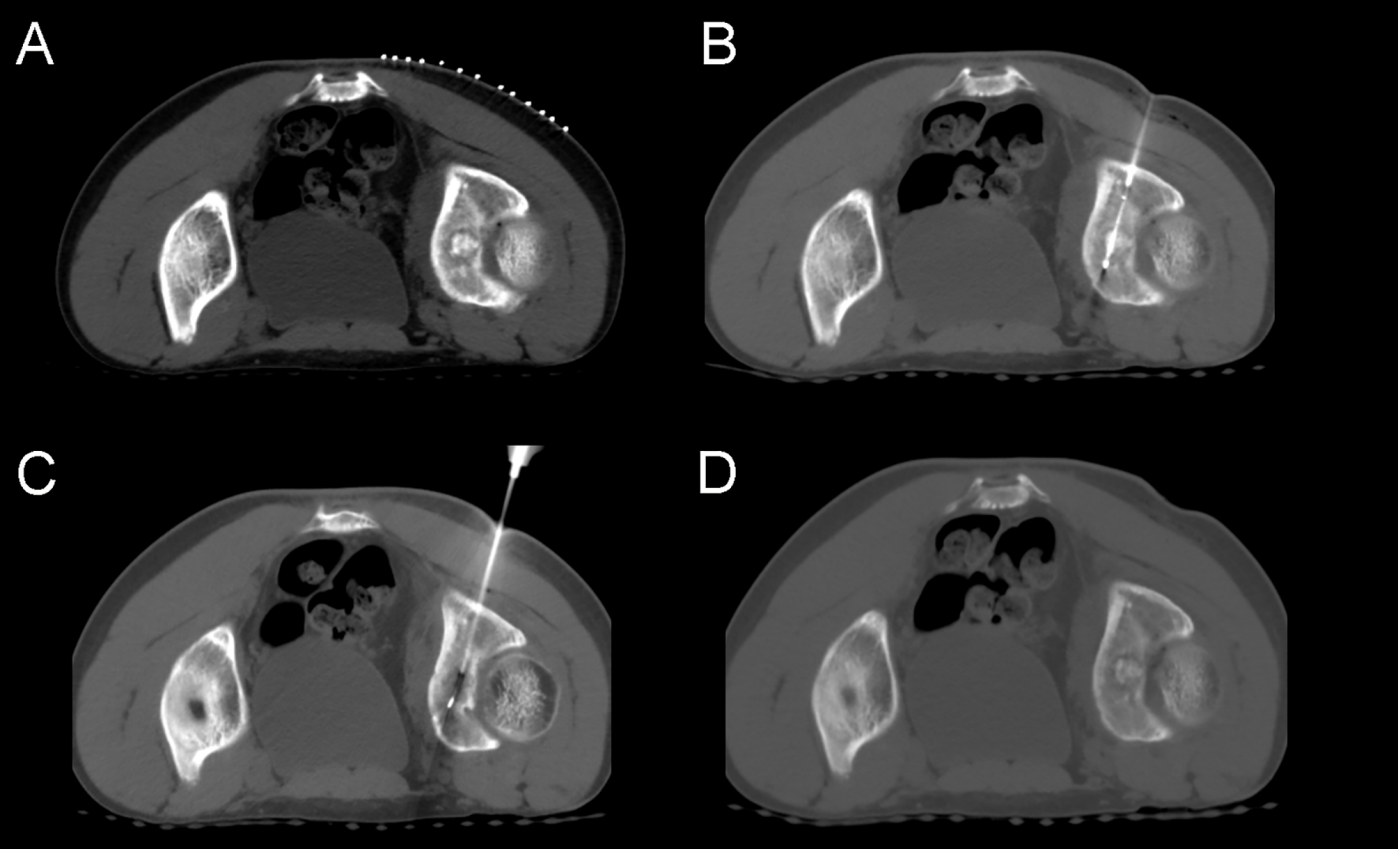
A 15-year-old male patient with left hip pain and claudication. (A) A large, round, high-density lesion was seen in the left acetabulum, surrounded by a circular hypodensity shadow. (B) Cryoprobe passed anteriorly through the lesion. (C) After two cycles of freezing, the lesion did not change significantly on CT images. No obvious ice ball could be seen. (D) After removal of the needle, the case was reviewed, and no other complications such as bleeding were found.

A total of 15 nidi from 15 patients underwent cryoablation. All patients’ mean age was 17.53 years (range 7–52), with eleven males and four females. Of all lesions, three were located in the vertebra (Figs. 1 and 2), seven in the femur’s neck, two in the tibia, and one in the acetabulum (Fig. 3), patella, or ilium. The mean age in the surgical operation group was 15.15 years (range 3–50), with 37 males and 16 females. Of all 53 lesions, 11 were located in the vertebra and 39 in the femur. Detailed clinical information was presented in Table 1.

### Treatment efficacy and follow-up

Only one patient in the cryoablation group underwent general anesthesia, and the rest were treated with percutaneous local anesthesia. Thirty-eight patients in the surgical resection group underwent general anesthesia, and fifteen patients were treated with epidural anesthesia (Table 2).

**Table 2:** Efficacy and complications of cryoablation versus surgical resection.

	Percutaneous cryoablation (*n* = 15)	Surgical resection (*n* = 53)	*p*-value
Days of hospitalization	6.87 ± 2.85 (3–12)	11.37 ± 4.91 (3–30)	0.001
Blood loss (ml)	2.27 ± 1.28 (2–5)	133.43 ± 306.00 (5–1700)	0.003
Anesthesia method			
Percutaneous local anesthesia	14	0	
General anesthesia	1	38	
Epidural anesthesia	0	15	
Technical success			
Yes	15	53	
No	0	0	
Pre-procedure VAS score (range)	6.33 ± 1.54 (3–8)	6.41 ± 1.00 (4–8)	
Post-procedure VAS score (range) (<1 month)	0.73 ± 0.70 (0–2)	0.78 ± 0.63 (0–2)	
Post-procedure VAS score (range) (>1 years)	0.13 ± 0.35 (0–1)	0.02 ± 0.14 (0–1)	
Follow-up duration (months) (range)			
Recurrences			
Yes	0	1	
No	15	52	
Complications			
Major	0	3	
Minor	3	28	
None	12	22	
Clinical success			
Yes	15	52	
No	0	1	

**Table 3:** Statistical analysis of VAS scores of cryoablation and surgery resection.

Procedure	Group	No. of patients	Mean	Std. deviation	*p*-value
Cryoablation	VAS pre-operation	15	6.33	1.54	<0.001
	VAS post-operation	15	0.73	0.70	
	VAS follow-up	15	0.13	0.35	<0.001
	VAS post-operation	15	0.73	0.70	
Surgery resection	VAS pre-operation	53	6.41	1.00	<0.001
	VAS post-operation	53	0.78	0.63	
	VAS follow-up	53	0.02	0.14	<0.001
	VAS post-operation	53	0.78	0.63	

**Notes.**

VAS, visual analog scale.

Technically, all patients successfully underwent cryotherapy or surgical resection as scheduled pre-procedure with a 100% success rate. Clinically, in the cryoablation group, we adopted the VAS score system to assess cryoablation and surgery’s therapeutic efficacy. All patients obtained pain relief immediately after the procedure, and most of them disappeared completely. Mean VAS score before cryoablation was 6.33 ±  1.54 and was 0.73 ±  0.70 after cryoablation within seven days, 0.13 ±  0.35 after cryoablation for more than twelve months. There were statistically significant differences in VAS scores between the pre-and post-operative groups (*p* < 0.001) (Tables 2 and 3). The median follow-up time in our study was 17 months (range 13–26).

All patients completed the surgery as planned in the surgical resection group and obtained rapid post-operative pain relief. Of all the patients who underwent surgery, fifteen underwent heterotopic, or allograft bone implantation and eleven underwent internal metal fixation. Seven patients underwent both bone grafting and internal fixation. The mean VAS score before surgery was 6.41 ±  1.00 and was 0.78 ±  0.63 after cryoablation within seven days, 0.02 ±  0.14 after surgery for more than twelve months. There were statistically significant differences in VAS scores between the pre-and post-operative groups (*p* <0.001) (Tables 2 and 3).

### Complications

In the cryoablation group, all patients did not experience any major complications during or after the treatment. Mild pain (VAS 1-2) was present in only six patients within seven days after the procedure, but most of these resolved independently in a short period of time, and only one case had constant mild discomfort. There was no incidence of infection or skin frostbite in any of the patients. In one of the three patients with spinal lesions, there was numbness in the left lower extremity after the procedure, which self-relieved within one week. Due to the inventive use of air for protection, although the lesion was close to the dural sac, while complete ablation was achieved, none of the patients experienced any irreversible nerve damage or other major complications (Figs. 1 and 2). None of the patients suffered significant scarring after cryoablation.

**Figure 4:**
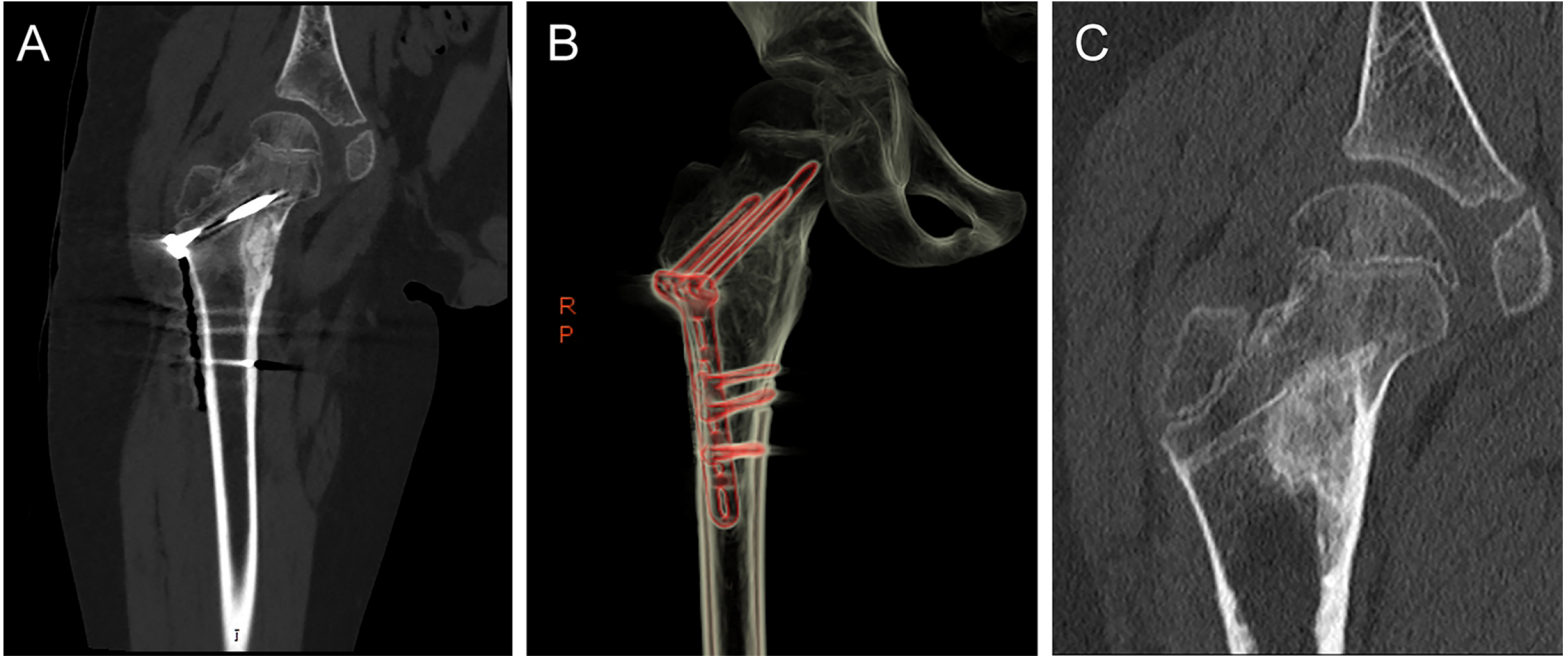
A 9-year-old boy with an osteoid osteoma of the right proximal femur who underwent surgical focal resection followed by bone grafting and internal fixation with plate screws. (A) Post-operative coronal CT image showed that the residual cavity of the lesion area was filled with dense allogeneic bone, and metal internal fixation screws and plates were visible. (B) Three-dimensional reconstruction image after screw and plate internal fixation. (C) One year after surgery, the CT scan was repeated, and the internal fixation device was removed, and the density and size of the lesion remained the same.

In the surgery group, three of the patients experienced massive bleeding (>500 ml), and two underwent blood transfusion therapy. Thirteen patients had minor complications, including mild pain (VAS 1-2), bloody fluid in the surgical area, and drains placement. Twenty patients were placed with drainage tubes after surgery, with an average drainage volume of 159.35 ml. Only one patient in the surgical resection group experienced a recurrence at 29 months postoperatively and underwent a second resection after that (Table 3). No complications such as osteonecrosis or fractures occurred in any of the patients. However, a total of 11 patients received internal fixation of screws and plates to prevent fractures due to the large size of the cavity or weight-bearing at the site (Fig. 4). Besides, all patients were left with visible scars after surgical resection.

Besides, in terms of length of hospitalization, the surgical group’s average length was 11.37 days, which was significantly longer than 6.87 days of the percutaneous cryoablation group (*p* = 0.001). Because it was a percutaneous minimally invasive treatment, intraoperative bleeding in the cryoablation group was minimal (<10 ml), while the average volume of the surgical group was about 133 ml.

## Discussion

Treatment for OO is mainly surgical excision, which can lead to recurrence if the tumor nest is not eradicated (Dookie & Joseph, 2020; Mallepally et al., 2020). While surgery can completely remove the tumor, it is more traumatic, recovery is slower, and may lead to major complications such as bleeding, bone ischemia, or fractures (Orth & Kohn, 2017). Therefore, more and more clinical methods of local ablation are now being used to treat the disease, which is less traumatic, has fewer complications, and has a good prognosis. At present, more reports were radiofrequency ablation and microwave ablation methods, both of which are thermal ablation and will cause pain during the procedure with local anesthesia (Pipola et al., 2020; Prud’homme et al., 2017; Tordjman et al., 2020). A recently published study by Joseph R et al. compared radiofrequency ablation and microwave ablation to treat OO. They found no significant differences between the two techniques regarding either long-term efficacy or complications (Reis, Chang & Sharma, 2020).

But for particular site lesions, cryoablation is sometimes more suitable. This study is a retrospective analysis with the primary objective of examining the efficacy and safety of cryoablation in treating OO in different body parts. We also included 53 patients treated with surgery at our institution during the same period as a control group to compare the differences between cryoablation and surgery in terms of treatment efficacy, trauma, recovery, and complications. As expected, all 15 patients who underwent cryoablation were successfully treated based on pre-and post-operative clinical data and medical imaging data. All of the patients’ pain relieved rapidly after the treatment was completed, and most of the patients’ pain disappeared entirely within a short period. At subsequent follow-up, all patients did not experience any relapse, and no major complications occurred. While the surgical group also achieved a 100% technical success rate, three patients experienced intraoperative bleeding, and one patient experienced recurrence, and a second surgery in the post-operative follow-up. According to our statistics, the surgery was significantly more traumatic than minimally invasive ablation, and the post-operative recovery was slower, and the length of the hospital stay longer. In particular, nine of the eleven patients with an OO in the vertebrae were treated with internal screw fixation while removing the lesion. Therefore, we believe that cryoablation is an effective and minimally invasive technique for the treatment of OO, with comparable efficacy to surgical resection, less trauma, fewer complications, and better safety. And our results are similar to the previous studies, where cryoablation had the same curative effect on patients as surgery (Basappa et al., 2019; Coupal et al., 2014; Miyazaki et al., 2018; Whitmore et al., 2016).

For OO, the purpose of thermal ablation is to kill the nidus cells while damaging the surrounding unmyelinated nerve fibers to eliminate the patient’s symptoms. Cryoablation inactivates tumor cells mainly through the following pathways: extracellular icing leading to a solution effect, intracellular icing, intra-microvascular thrombosis, and it may lead to apoptosis. Quick thawing of tissues allows fluid to enter the damaged cell membrane leading to cell rupture, and shear forces of the recrystallization process may further injure cells (Erinjeri & Clark, 2010). Although microwave and radiofrequency ablation can also be very effective, we believe that cryoablation’s main advantages include the following aspects: first, patients experience less pain during and after cryoablation due to the intrinsic analgesic properties of ice (Callstrom & Kurup, 2009). Second, bone cells are more sensitive and conducive to freezing, whereas the efficiency of thermal conduction of radiofrequency ablation may be affected by the bone tissue (Kuylenstierna & Lundquist, 1982). Besides, in the case of OO in specific areas such as the vertebral body, the injection of air can effectively isolate the damage of freezing to the adjacent vital structures, and air does not adequately insulate the transfer of thermal effects in radiofrequency and microwave ablation. It is noteworthy that in the follow-up of a case of the vertebral lesion where cryoablation was performed (Fig. 2), it was found that the reactive osteosclerosis around the original lesion also basically returned to normal spontaneously, and the mechanism of its recovery needs to be further investigated.

We have successfully cured three patients with an OO in the cryoablation group occurring in the vertebral body or adnexa, ranging in age from 11 to 35. These patients were treated with consideration of complete inactivation of the tumor nest and the protection of the dura mater, spinal cord, and nerve roots. Therefore, for better outcomes and fewer complications, our approach is to push another fine needle into the epidural space while freezing and inject a volume of 60–80 ml filed air into it to push the dural sac as far out of the treatment range of the freezing probe as possible and insulate it from extreme hypothermia. With this approach, none of our patients has experienced nerve root damage or other serious complications, and the treatment has been very effective. We did not find any descriptions of experiences similar to our approach in the relevant literature. Clinically, during the ablation of lung lesions close to areas such as the pleura or heart, we also take an active pneumothorax to protect adjacent normal structures from damage. During cryoablation of subcutaneous soft tissue lesions, subcutaneous emphysema can also be created to prevent skin frostbite.

None of the patients had major complications in the cryoablation group, and only two patients had mild post-operative distention in the treated area. The symptom dissolved after giving routine post-operative care, such as anti-inflammatory and analgesic. The complications of cryoablation of OO are closely related to the size and location of the lesion. If the lesion was located in a weight-bearing area, such as the femur or tibia, it could lead to osteonecrosis or fracture; if the lesion was located in a joint area such as the acetabulum, it might lead to synovitis, tendonitis, or dysfunction; if the lesion was close to the spinal cord or nerve roots, it might lead to nerve damage and associated symptoms. Also, there are frequent complications, such as hemorrhage, hematoma, and skin frostbite. For example, Whitmore et al. reported 23 cases of cryoablation for OO. They found only six cases with minor complications, including three cases of mild skin blistering, one case of numbness of the nerves on the back of the foot and the toes, and two cases of mild pain and weakness in the treated area (Whitmore et al., 2016). In our experience, since the cryoprobe cannot penetrate the bone directly, an 11G coaxial T-Lok bone marrow biopsy needle system was firstly used to penetrate the osteocortex, which has sound temperature isolation and insulation during the freezing process. Therefore, no obvious skin frostbite or blistering was seen in any of our patients. In particular, in three cases of OO occurring in the spine, due to our innovative use of a 19G fine needle to inject air into the epidural space, none of the symptoms associated with nerve injury or pressure occurred, except for a slight post-operative swelling sensation in the lumbar region. For the nidus occurring on the acetabulum, we treated it by adjusting the angle cryoprobe to avoid the articular cartilage. Due to the lesion’s greater size, we performed two treatment cycles with 15 min of freezing each cycle. Postoperatively, the patient recovered well, and no joint dysfunction or developmental abnormalities were noted (Fig. 3). Another aspect of cryoablation’s marked superiority over surgical resection is its minimally invasive nature, with minimal incisions and no scars left behind. However, surgical resection is an open procedure that leaves large scars, causing psychological distress for adolescents.

Another potential advantage of cryoablation over surgery is freezing only for nidus, which will obtain an excellent result without damage to the bony structure. The most nidus size is less than 1 cm, so one 17G probe is enough for the treatment. The critical point of cryoablation is to eradicate the nidus. The surrounding sclerotic bone is a secondary reaction caused by the nest’s stimulation, so freezing of the surrounding is not necessary. However, surgical resection must excavate the sclerotic bone to find the nidus to achieve the treatment effect, but the nidus was always difficult to identify during osteotomy, resulting in incomplete resection and post-operative recurrence; and surgical osteotomy destroys the bone structure and may cause fractures (Payo-Ollero et al., 2020). Therefore, the lesion’s surgical removal may be actually larger than what needs to be treated, bringing added trauma to the patient. However, a reduction in the extent of resection may, in turn, lead to an increase in the recurrence rate. These are issues that need to be validated and discussed in more studies in the future. For the lesions in the weight-bearing areas such as the femoral neck, the surgical treatment will be limited, cut through the hardened bone structure to destroy the nidus. There is a risk of associated fractures after the surgery. Additionally, periarticular lesions, such as at the femoral neck, tumor nests were often located within the joint capsule. The cryoablation probe will not damage the joint capsule’s healthy structure, which is different from surgical resection (Germann et al., 2020; Yano, Kaneshiro & Sakanaka, 2020).

All patients in this study were treated under CT guidance as in previous studies (Coupal et al., 2014; Tordjman et al., 2020; Wu et al., 2011). The main advantage of CT is the rapid imaging speed and better display of bone density. However, CT has limited resolution of soft tissues such as blood vessels, nerves, and muscles. In recent years, MRI has been increasingly used in the guidance of percutaneous interventional procedures, including prostate cancer and renal cell carcinoma, and its main advantage lies in its ability to detect soft tissues inside and around the lesion (Bhagavatula et al., 2020; Mathew & Oto, 2017). The display of structures allows for more precise localization of lesions and less damage to adjacent critical structures (Cazzato et al., 2018). Due to the high density of bone, the ice ball formed during the freezing of OO does not appear clearly on CT, but since the ice ball appears as a signal-free zone on MRI, the MRI image may clearly delineate the extent of the ice ball. Our hospital has just introduced the country’s first 3.0 T high-field magnetic resonance guidance system. In future work, we will attempt MR-guided cryoablation for OO.

In monitoring CT-guided cryoablation, it is essential to evaluate the effective killing range and the extent of damage to surrounding structures. On CT images, the extent of the iceball cannot be clearly shown in the bone tissue. Suppose the lesion is deeply located and the iceball is completely within the bone tissue. In that case, it can only be determined by the freezing parameters of the cryoprobe and the surgeon’s experience. But in this case, there is no need to consider the damage to the surrounding soft tissues since the surrounding area is surrounded by bone. If the lesion is superficial, the iceball may cover the surrounding soft tissues, and the relationship between the low-density iceball in the soft tissues and the surrounding structures can be visualized by CT to minimize damage to essential structures. The temperature at the edge of the iceball is 0 degrees, and the effective damage zone of freezing is usually 5 mm or more from the edge of the iceball. For some of the lesions that are close to the bone cortex, we can indeed achieve ablation by placing the probe close to the osteocortex rather than penetrating the lesion. This approach’s choice is based on the distance of the lesion from the long axis of the probe, and as long as it is within the effective killing range of the probe, it is possible to inactivate the nest. For the duration of cryoablation, we routinely use two cycles of treatment. Each cycle consists of 10 min of argon freezing and 3 min of helium rewarming to achieve complete inactivation of the tumor nest.

There are several limitations to this study. First, due to the retrospective property, there was no rigorous follow-up process, and the time point of follow-up was not fixed, and some patients were excluded because they did not have long-term follow-up data. Second, not all patients had undergone puncture biopsies and pathological verification, and the diagnosis of OO was based on clinical symptoms and radiographic manifestations. Third, the number of recruited cases we collected is still limited, and more clinical trials are still needed in the future to validate the efficacy and safety of cryoablation of OO in different body sites.

## Conclusions

We retrospectively analyzed the technique, efficacy, and safety of using cryoablation to treat osteoid osteoma cases at our institution and compared it to the patients treated surgically at the same time. The results were encouraging, and we believe that cryoablation is a minimally invasive, safe, and effective treatment that is fully comparable to surgery.

##  Supplemental Information

10.7717/peerj.10724/supp-1 Supplemental Information 1 Raw data of patients who underwent surgical procedures

10.7717/peerj.10724/supp-2 Supplemental Information 2 Raw data of patients who underwent cryoablation

## Additional Information and Declarations

### Data Availability

The following information was supplied regarding data availability: Raw clinical data are available in the Supplemental Files.

## References

[ref-1] Agashe M, Vaidya S, Dhamele J, Chauhan H, Naik P, Nagda T (2020). CT-guided percutaneous drilling of osteoid osteoma: a safe, minimally invasive and cost-effective method. Indian Journal of Orthopaedics.

[ref-2] Atesok KI, Alman BA, Schemitsch EH, Peyser A, Mankin H (2011). Osteoid osteoma and osteoblastoma. The Journal of the American Academy of Orthopaedic Surgeons.

[ref-3] Basappa E, Rabang J, Anderson W, Richardson R, Scott R (2019). CT-guided percutaneous cryoablation of an osteoid osteoma of the rib. Radiology Case Reports.

[ref-4] Bhagavatula SK, Tuncali K, Shyn PB, Levesque VM, Chang SL, Silverman SG (2020). Percutaneous CT- and mri-guided cryoablation of CT1 renal cell carcinoma: intermediate- to long-term outcomes in 307 patients. Radiology.

[ref-5] Callstrom MR, Kurup AN (2009). Percutaneous ablation for bone and soft tissue metastases–why cryoablation?. Skeletal Radiology.

[ref-6] Cazzato RL, Garnon J, Shaygi B, Tsoumakidou G, Caudrelier J, Koch G, Gangi A (2018). How to perform a routine cryoablation under MRI guidance. Topics in Magnetic Resonance Imaging.

[ref-7] Coupal TM, Mallinson PI, Munk PL, Liu D, Clarkson P, Ouellette H (2014). CT-guided percutaneous cryoablation for osteoid osteoma: initial experience in adults. AJR American Journal of Roentgenology.

[ref-8] De Filippo M, Russo U, Papapietro VR, Ceccarelli F, Pogliacomi F, Vaienti E, Piccolo C, Capasso R, Sica A, Cioce F, Carbone M, Bruno F, Masciocchi C, Miele V (2018). Radiofrequency ablation of osteoid osteoma. Acta Bio-Medica: Atenei Parmensis.

[ref-9] Dookie AL, Joseph RM (2020). Osteoid Osteoma.

[ref-10] Erinjeri JP, Clark TW (2010). Cryoablation: mechanism of action and devices. Journal of Vascular and Interventional Radiology.

[ref-11] Esteban Cuesta H, Martel Villagran J, Bueno Horcajadas A, Kassarjian A, Rodriguez Caravaca G (2018). Percutaneous radiofrequency ablation in osteoid osteoma: tips and tricks in special scenarios. European Journal of Radiology.

[ref-12] Germann T, Weber M-A, Lehner B, Kintzele L, Burkholder I, Kauczor H-U, Rehnitz C (2020). Intraarticular osteoid osteoma: MRI characteristics and clinical presentation before and after radiofrequency ablation compared to extraarticular osteoid osteoma. RoFo: Fortschritte auf dem Gebiete der Rontgenstrahlen und der Nuklearmedizin.

[ref-13] Jannelli G, Moiraghi A, Schaller K, Tessitore E (2020). Navigation assisted tubular resection of lumbar osteoid osteoma: how I do it. Acta Neurochirurgica.

[ref-14] Kuylenstierna R, Lundquist PG (1982). Bone destruction by direct cryoapplication: a temperature study in rabbits. Cryobiology.

[ref-15] Lindquester WS, Crowley J, Hawkins CM (2020). Percutaneous thermal ablation for treatment of osteoid osteoma: a systematic review and analysis. Skeletal Radiology.

[ref-16] Mallepally AR, Mahajan R, Pacha S, Rustagi T, Marathe N, Chhabra HS (2020). Spinal osteoid osteoma: surgical resection and review of literature. Surgical Neurology International.

[ref-17] Mathew MS, Oto A (2017). MRI-guided focal therapy of prostate cancer. Future Oncology.

[ref-18] Miyazaki M, Saito K, Yanagawa T, Chikuda H, Tsushima Y (2018). Phase I clinical trial of percutaneous cryoablation for osteoid osteoma. Japanese Journal of Radiology.

[ref-19] Orth P, Kohn D (2017). Diagnostics and treatment of osteoid osteoma. Der Orthopade.

[ref-20] Payo-Ollero J, Moreno-Figaredo V, Llombart-Blanco R, Alfonso M, San Julián M, Villas C (2020). Osteoid osteoma in the ankle and foot. An overview of 50 years of experience. Foot and Ankle Surgery.

[ref-21] Pipola V, Tedesco G, Spinnato P, Facchini G, Gala RB, Bandiera S, Brodano GB, Terzi S, Ghermandi R, Evangelisti G, Ricci A, Griffoni C, Pezzi A, Gasbarrini A (2020). Surgery versus radiofrequency ablation in the management of spinal osteoid osteomas: a spine oncology referral center comparison analysis of 138 cases. World Neurosurgery.

[ref-22] Prud’homme C, Nueffer JP, Runge M, Dubut J, Kastler B, Aubry S (2017). Prospective pilot study of CT-guided microwave ablation in the treatment of osteoid osteomas. Skeletal Radiology.

[ref-23] Reis J, Chang Y, Sharma AK (2020). Radiofrequency ablation vs microwave ablation for osteoid osteomas: long-term results. Skeletal Radiology.

[ref-24] Ringe KI, Panzica M, Von Falck C (2016). Thermoablation of bone tumors. RoFo: Fortschritte auf dem Gebiete der Rontgenstrahlen und der Nuklearmedizin.

[ref-25] Santiago E, Pauly V, Brun G, Guenoun D, Champsaur P, Le Corroller T (2018). Percutaneous cryoablation for the treatment of osteoid osteoma in the adult population. European Radiology.

[ref-26] Takahashi H, Berber E (2020). Role of thermal ablation in the management of colorectal liver metastasis. Hepatobiliary Surgery and Nutrition.

[ref-27] Tomasian A, Cazzato RL, Auloge P, Garnon J, Gangi A, Jennings JW (2020). Osteoid osteoma in older adults: clinical success rate of percutaneous image-guided thermal ablation. Clinical Radiology.

[ref-28] Tomasian A, Jennings JW (2019). Hot and cold spine tumor ablations. Neuroimaging Clinics of North America.

[ref-29] Tordjman M, Perronne L, Madelin G, Mali RD, Burke C (2020). CT-guided radiofrequency ablation for osteoid osteomas: a systematic review. European Radiology.

[ref-30] Whitmore MJ, Hawkins CM, Prologo JD, Marshall KW, Fabregas JA, Yim DB, Monson D, Oskouei SV, Fletcher ND, Williams RS (2016). Cryoablation of osteoid osteoma in the pediatric and adolescent population. Journal of Vascular and Interventional Radiology.

[ref-31] Wu B, Xiao Y-Y, Zhang X, Zhao L, Carrino JA (2011). CT-guided percutaneous cryoablation of osteoid osteoma in children: an initial study. Skeletal Radiology.

[ref-32] Wu H, Lu C, Chen M (2017). Evaluation of minimally invasive laser ablation in children with osteoid osteoma. Oncology Letters.

[ref-33] Yano K, Kaneshiro Y, Sakanaka H (2020). Arthroscopic excision for intra-articular osteoid osteoma of the olecranon fossa: a case report and literature review. Case Reports in Orthopedics.

[ref-34] Young S, Golzarian J, Anderson JK (2019). Thermal ablation of t1a renal cell carcinoma: the clinical evidence. Seminars in Interventional Radiology.

